# Reduced Susceptibility to the Dunning–Kruger Effect in Autistic Employees

**DOI:** 10.1002/aur.70139

**Published:** 2025-11-12

**Authors:** Lorne M. Hartman, Harley Glassman, Braxton L. Hartman

**Affiliations:** ^1^ Organization Studies, Schulich School of Business York University Toronto Canada; ^2^ Department of Psychology Toronto Metropolitan University Toronto Canada; ^3^ Department of Psychology York University Toronto Canada

**Keywords:** autism, cognitive bias, metacognitive awareness, neurodiversity

## Abstract

Evidence indicates that autistic individuals are less susceptible to social influence and cognitive biases than non‐autistic individuals. However, no studies have been conducted on the Dunning–Kruger effect (DKE) in autism. The DKE is a cognitive bias in which people with limited expertise in a specific domain overestimate their abilities. The purpose of this study is to compare autistic and non‐autistic employees' self‐assessments of their performance with their objective performance on a popular performance‐based measure of analytic thinking disposition, the CRT (cognitive reflection test). After completing the task, no feedback or clues were provided regarding how well they performed. Participants were then asked to estimate how many questions they answered correctly and compare their performance to other participants by estimating the percentage of peers they outperformed. Results indicated asymmetric calibration of actual versus estimated CRT performance in autistic employees: In the low‐performance group, autistic participants overestimated their abilities less than non‐autistic participants. However, in the high‐performance group, autistic participants underestimated their abilities more than non‐autistic participants. Reduced susceptibility to the DKE highlights potential benefits of autistic employees in the workplace. Theoretical and practical implications consider the intersection of metacognitive awareness, autism, and the DKE in an organizational context.

**Summary**

- The purpose of this study is to compare autistic and non‐autistic employees' assessments of their performance with their objective performance on a popular performance‐based measure of analytic thinking disposition, the CRT (cognitive reflection test).
- While low‐performing employees often overestimate their abilities (a bias known as the Dunning–Kruger effect), autistic employees appear less susceptible to this distortion.
- This study contributes to a growing body of empirical evidence highlighting potential advantages of autistic employees in the workplace.
- The results of this study also have implications for human resource practices in hiring, onboarding, and managing autistic employees.

## 1 Introduction

Not knowing the scope of your own ignorance is part of the human condition. The problem is that we see it in other people, but we don't see it in ourselves. The first rule of the Dunning–Kruger club is you don't know you're a member of the Dunning–Kruger club.

David Dunning (Resnick 2019).

Autism is a lifelong neurodevelopmental condition characterized by (1) difficulties in social interaction and communication and (2) restricted and repetitive patterns of behavior (American Psychiatric Association 2013). Global prevalence varies, but recent estimates indicate that approximately 1% of adults (1.8% in males and 0.2% in females) are diagnosed with autism (Zeidan et al. 2022). It is important to note that autism is a “spectrum” with many dimensions, and the complexities of navigating the world as an autistic^1^ individual are different for everyone. This means that autistic individuals exhibit a wide range of abilities and challenges.

Many autistic individuals have a strong interest in employment and possess relevant employment strengths, including above‐average abilities and skills, some of which are in high demand but scarce in the job market (Doyle 2020). However, autistic people experience disproportionately high rates of unemployment or underemployment (Hurley‐Hanson and Giannantonio 2016).

Despite the challenges and barriers, employers are increasingly interested in leveraging the potential benefits of autistic employees in the workplace (Austin and Pisano 2017). Research is beginning to elucidate ways in which certain differences between autistic and non‐autistic people, rather than being deficits, might prove to be advantages for autistic employees in organizations. In particular, a growing body of empirical evidence suggests that autistic individuals are less susceptible to social influence and cognitive biases than non‐autistic individuals (see Rozenkrantz et al. 2021, for a review). For example, in comparison to non‐autistic controls, autistic participants are (1) less affected by the “sunk cost” bias, i.e., they are more inclined to focus on current and future avoidable costs rather than irrecoverable sunk costs when making decisions (Fujino et al. 2017; Rogge 2021), (2) less prone to “framing effects,” making them less likely to prefer one of two mathematically identical options based on whether it is presented as a gain or a loss (De Martino et al. 2008; Shah et al. 2016), (3) tend to be more deliberative in reasoning, relying less on intuition or emotion (Brosnan and Ashwin 2023; Brosnan et al. 2014; Brosnan et al. 2016; Farmer et al. 2017; Levin et al. 2015), (4) less susceptible to the “conjunction fallacy,” that is, the inclination to favor multiple specific conditions over a single underlying cause driven by the perceived likelihood of specific conditions due to the prominence of representative information (Morsanyi et al. 2010), (5) less likely to make moral judgments about an action based on an analysis of a person's intention or character and more likely to rely on negative outcomes of the action (Komeda et al. 2016; Moran et al. 2011), (6) less influenced by social pressures and concerns about managing their reputation (Frith and Frith 2011; Izuma et al. 2011), (7) less prone to bias when revising self‐referential beliefs (Kuzmanovic et al. 2019), (8) more likely to accept offers deemed to be unfair but economically advantageous (Jin et al. 2020; Wang et al. 2019); (9) less susceptible to implicit bias based on race and gender (Birmingham et al. 2015; Kirchner et al. 2012); (10) less susceptible to the bystander effect and less likely to espouse false beliefs about whether they were influenced by the presence of others when deciding to take action (Hartman et al. 2023); (11) less vulnerable to “moral disengagement,” a cognitive process of reframing unethical behavior as morally acceptable without altering the behavior itself or the underlying moral standards (Hartman and Hartman 2024a); and (12) less influenced by potential biasing social information when making decisions (Forbes et al. 2024; Sevgi et al. 2020).

These findings highlight the potential benefits of autistic employees in the workplace.^2^ For example, because autistic employees are less susceptible to the bystander effect (Hartman et al. 2023), they are more likely to say something or do something when they see something wrong happening in an organizational context, whether it is gross misconduct on the part of a manager or an error in a training manual. By bringing attention to operational inefficiencies or ethical transgressions, autistic employees may contribute to improvements in firm performance.

However, there have not been any studies on the Dunning–Kruger effect (DKE) in autism. The DKE is a cognitive bias in which people with limited competence in a particular domain overestimate their abilities (Kruger and Dunning 1999). Some researchers include a “metacognitive” component in their definition, that is, those who are incompetent tend to be ignorant of their incompetence, presumably because they lack the metacognitive ability to become aware of their incompetence. This is sometimes called the “dual burden” account because low performers face two challenges: (1) a lack of skill and (2) an inability to recognize this deficiency. Additionally, some investigators consider the reverse effect, where highly skilled individuals underestimate their abilities compared to others.

Understanding the DKE is highly relevant to understanding workplace dynamics, organizational behavior, and human resource management because it sheds light on how individuals' perceptions of their own competence influence decision‐making, team dynamics, and leadership. Overconfidence in one's own judgment can have disastrous consequences. High rates of entrepreneurial failure, global stock market crashes, the explosion of the Space Shuttle Challenger, and the nuclear accident at Chernobyl have all been blamed on overconfidence (Moore and Healy 2008). For example, overconfident employees may dominate discussions or decision‐making processes, potentially stifling input from more competent team members who undervalue their own contributions (Dhannur and Kusane 2023). This imbalance can lead to poor team performance, as decisions are driven by confidence rather than competence (Klabzuba and Mumford 2011). Leaders exhibiting the DKE may fail to recognize their own shortcomings, resulting in ineffective leadership styles or misguided strategic decisions. These leaders might also resist feedback or dismiss input and advice from others, creating a toxic work environment (Cohee and Barnhart 2024).

While no studies have linked autism to the DKE, related research on certain cognitive and behavioral tendencies associated with autism provides a strong theoretical basis for exploring this hypothesis. For example, despite a widespread and longstanding assumption that autistic people lack self‐insight, research indicates that (1) autistic people are less susceptible to false beliefs about themselves, for example, they are more likely to acknowledge the influence of other people being present when deciding whether to intervene in a bystander situation (Hartman et al. 2023); (2) they are less biased when updating self‐referential beliefs (Kuzmanovic et al. 2019); (3) and they are less likely to endorse self‐serving cognitive biases to minimize negative self‐evaluation (Hartman and Hartman 2024a). In addition, autistic people are less biased by prior experience and, as a result, can perceive the world and themselves more accurately (Happé and Frith 2006; Pellicano and Burr 2012). These findings are consistent with other reports indicating no association between autistic traits and metacognitive accuracy (Brewer et al. 2022; Embon et al. 2023). For example, autistic individuals show significant levels of understanding of their personality across different operationalizations of self‐insight (Schriber et al. 2014). As a result of the reduced influence of social desirability biases such as a desire to appear knowledgeable or to gain approval (e.g., Izuma et al. 2011), autistic individuals may be less likely to overestimate their competence to “fit in” or impress others.^3^ Because autistic people often focus on specific details (e.g., Alink and Charest 2020), they may be less likely to make broad generalizations about their abilities and, in turn, be more realistic about their competencies in a certain domain. In addition, evidence indicates a strong preference for honesty and factual correctness, a truth‐telling tendency in autism (e.g., Baron‐Cohen 2008), and therefore, autistic people may be uncomfortable overestimating their abilities, even in social contexts where modest exaggeration is common. Research also suggests that autistic individuals experience heightened self‐consciousness and anxiety about performance (e.g., Maisel et al. 2016), which might make them more cautious in evaluating their performance or more measured when evaluating their skills. Together, these findings suggest that autistic people may be more willing to admit gaps in their understanding and knowledge, which would counteract the overestimation typically observed in the DKE.

While a few studies have reported diminished metacognitive accuracy in judgments‐of‐performance (JOP) tasks in autism (e.g., Furlano and Kelley 2019; Furlano et al. 2015), these studies were conducted with children and adolescents. One study conducted with adults measured metamemory monitoring ability using a feeling‐of‐knowing task and found that autistic participants made significantly more errors of the under‐confident type but not the over‐confident type (Grainger et al. 2014). Beyond the studies already summarized, a more nuanced picture of metamemory in autism emerges when considering different metacognitive measures such as judgments‐of‐learning (JOL), feelings‐of‐knowing (FOK), and judgments‐of‐confidence (JOC). Each of these paradigms taps distinct aspects of monitoring: JOLs assess prospective predictions about future recall, FOKs capture retrospective predictions about the likelihood of recognizing unrecalled information, and JOCs index confidence in already retrieved responses. Existing evidence suggests a mixed profile in autistic populations. For instance, some studies report relatively intact confidence judgments (JOCs) in recognition tasks (Chua et al. 2009) while others highlight reduced calibration in JOLs under certain conditions (Grainger et al. 2016a, 2016b; Wojcik et al. 2013), indicating difficulties in accurately predicting future memory performance. Research on FOK in autism is more limited, but preliminary findings suggest potential challenges in monitoring retrieval processes (e.g., Sodian et al. 2015).

Importantly, as with JOP, much of this work has been conducted with children and adolescents, raising questions about developmental generalizability to adults on the spectrum. Grainger et al. (2016a) and Grainger et al. (2016b) found that autistic adults show typical JOL accuracy on both cue‐alone and cue‐target tasks, suggesting prospective judgments of learning may be preserved. However, Cooper et al. (2016) show that in monitoring tasks that involve distinguishing perceived vs. imagined sources, autistic adults exhibit lower metamemory sensitivity and confidence when having to monitor internal vs. external information. Meanwhile, Justus A. et al. (2021) observed that, in tasks involving context memory and confidence judgments, many aspects were comparable between adults with and without autism, though certain demanding conditions still revealed differences. Taken together, these findings suggest that metacognitive accuracy in autism may not be uniformly impaired or preserved, but instead may vary depending on the measure employed, the age of participants, and the demands of the task (Carpenter and Williams 2023). Future research directly comparing JOL, FOK, and JOC performance in adults will be critical to clarifying whether observed differences reflect developmental change, methodological artifacts, or genuine domain‐specific variability.

However, based on the relevant behavioral and metacognitive literature at this time, we expect that any early impairments in metacognitive accuracy in JOP tasks among autistic children will lessen with age, resulting in a reduced (but not absent) DKE in autistic adults compared to non‐autistic adults. This account has been previously suggested by others (e.g., Carpenter and Williams 2023) and is consistent with the developmental trajectory of impression management, theory of mind, and false belief understanding in autism (Happé 1995; Hull et al. 2017; Scheeren et al. 2013; Sommer et al. 2018).

The purpose of this study is to compare autistic and non‐autistic employees' assessments of their performance with their objective performance on a popular performance‐based measure of analytic thinking disposition, the CRT (cognitive reflection test). The CRT is believed to measure how well people reason and make decisions when solving problems. Participants must suppress an intuitively appealing incorrect response by engaging in further cognitive reflection, which could lead to a correct response (Frederick 2005). For instance, in the “bat and ball” problem, people attempt to solve this verbal problem: “A bat and a ball cost $1.10 in total. The bat costs $1.00 more than the ball. How much does the ball cost?” (Frederick 2005, 27). Although the correct response is “5 cents,” many participants give the response “10 cents,” which seems to pop into mind effortlessly. As a result, a property of the CRT is that for each item, almost all participants produce either the normatively correct response (which we will call “Analytical”) or a typical incorrect (i.e., “Intuitive”) response. “Atypical” responses are neither Analytical nor Intuitive.

Although not related to the present investigation, it is important to point out that previous studies investigating differences between autistic and non‐autistic individuals in performance on the CRT have produced mixed results. Some studies have shown that autistic participants give fewer intuitive and a higher proportion of analytic responses than non‐autistic participants (Brosnan et al. 2016; Brosnan et al. 2017; Brosnan and Ashwin 2023). Other findings suggest that performance on the CRT is not related to autism, particularly when the effects of cognitive ability are considered (Taylor et al. 2022; Morsanyi and Hamilton 2023). However, the current study is not intended to replicate or extend previous work regarding cognitive reflection in autism. We are interested in whether there are differences between autistic and non‐autistic participants in accurately estimating their abilities, using participants' scores on the CRT as the parameter of interest.

After completing the CRT task, no feedback or clues are provided as to how well they performed. Participants are asked to estimate how many questions they answered correctly and compare their performance to other participants by estimating the percentage of peers they outperformed. The main dependent variable is the difference between each participant's self‐assessment and their objective performance. We expect that autistic participants will be more analytical in their approach to the task and therefore should be better at recognizing their bias. Accordingly, among poor performers, we predict better calibration of actual versus estimated CRT performance by autistic participants. Among skilled performers, we predict the reverse effect, that is, autistic participants will underestimate their abilities more than non‐autistic participants.

### 1.1 Community Involvement

An important aspect of this study is that one of the authors, an autistic graduate student in neuroscience, was involved throughout the process from developing the research question, designing the methodology, analyzing the data, and writing up the findings. An additional feature of this study is that several of the autism support organizations that played a key role in soliciting autistic participants also helped to review the study protocol to ensure the research would appropriately represent the population.

## 2 Methods

### 2.1 Participants

We recruited our autistic participants through autism and disability support organizations in Canada, and the U.S. Diagnostic status was self‐reported. Some of the non‐autistic participants (e.g., family members and agency staff) were recruited this way, and the remainder of the non‐autistic participants were recruited through social media. A power analysis using G*Power (Faul et al. 2007) indicated that a sample size of 80 participants would be required to detect a moderately large effect size (*F* = 0.37, corresponding to *η* ^2^ = 0.12) with 90% power (*α* = 0.05) for a two‐way ANOVA. This estimate was based on a similar study by Bastan et al. (2025), who reported a comparable effect of autism diagnosis (*η* ^2^ = 0.12) on subjective cognitive style (analytical vs. intuitive). Given the conceptual overlap with our investigation of CRT performance and self‐estimation across diagnostic groups and tertiles, we set a target sample size of 100 to ensure sufficient power and account for potential data loss.

Participants were asked to click on a link to access the Qualtrics site for the study where they provided informed consent and completed demographic questions: age, sex, education level, employment status, whether they had been diagnosed as autistic, and, if autistic, the age they were diagnosed and type of professional that provided the diagnosis, that is, physician, psychiatrist, psychologist, social worker, occupational therapist, or other. After removing three autistic and two non‐autistic participants who had missing data, the resulting sample consisted of 53 autistic adults (average age = 32.2 years; 14 males and 39 females, mean SATQ score = 2.1) and 47 non‐autistic adults (average age = 38.7 years; 14 males and 33 females; mean SATQ score = 1.6). A chi‐square test indicated that the gender distribution of the two groups was not significantly different. In the autistic sample, participants shared further details regarding their diagnosis, including the age at which they were diagnosed (*M* = 26.5 years, SD = 12.73, min = 2, max = 53) and the person providing the diagnosis (Physician = 51%; Psychologist = 38%; Other, for example, Nurse Practitioner or Social Worker = 11%). Four participants (1 autistic and 3 non‐autistic) were not working at the time of the study.^4^ The majority, however, were employed full‐time (more than 30 h per week). The remaining participants were employed part‐time (10–30 h per week) or occasionally (less than 10 h per week). Further details regarding demographic characteristics of participants are provided in Table 1 and the Supporting Information (Appendix C, Figures S1 and S2).

**TABLE 1:** Sample characteristics.

	Autistic	Non‐autistic	Test statistic	*p*	Effect size
Age *M* (SD)	32.2 (9.7)	38.7 (14)	*t* = −2.63	0.01	0.27
Sex *N* (%)			*x* ^2^ < 0.01	1	< 0.001
Female	39 (73.1)	33 (72.7)			
Male	14 (26.9)	14 (27.3)			
Education *N* (%)			*x* ^2^ = 3.26	0.66	0.18
Primary	3 (5.8)	0 (0)			
Secondary	9 (15.4)	10 (18.2)			
Post‐secondary (BA)	25 (48.1)	22 (47.7)			
Master's degree	10 (19.2)	8 (18.2)			
Doctoral degree	1 (1.9)	2 (4.5)			
Other	5 (9.6)	5 (11.4)			
Employment status *N* (%)			*x* ^2^ = 4.51	0.21	0.21
Full Time	29 (55.8)	25 (56.8)			
Part‐Time	8 (15.4)	11 (25.0)			
Occasional	15 (28.8)	8 (18.2)			
Unemployed	1 (1.9)	3 (6.4)			
SATQ *M* (SD)	2.1 (0.3)	1.6 (0.5)	*t* = 6.04	< 0.01	0.62
CRT *M* (SD)					
Analytical	3.4 (2.1)	2.7 (2.1)	*t* = 1.54	0.13	0.16
Intuitive	1.7 (1.7)	2.5 (1.9)	*t* = −2.05	0.04	0.21
Atypical	0.9 (1.2)	0.1 (0.9)	*t* = 0.51	0.61	0.05
Estimated score	4.15 (1.7)	4.49 (1.59)	*t* = −1.03	0.31	0.21
Difference score	0.75 (2.07)	1.85 (2.02)	*t* = −2.67	0.01	0.54

*Note:* Effect sizes (*η* ^2^) for small, medium, and large effects are 0.01, 0.06, and 0.14.

Abbreviations: CRT: cognitive reflection test; SATQ: Sub‐Threshold Autistic Traits Questionnaire; SD: standard deviation.

### 2.2 Materials and Procedure

To confirm whether the autism group is higher in autistic traits than the non‐autistic group, participants completed the Subthreshold Autistic Trait Questionnaire (SATQ; Kanne et al. 2012). This 24‐item self‐report questionnaire assesses broad phenotypic autistic traits with strong psychometric support including structural validity (e.g., Cronbach's alpha coefficient = 0.73, test–retest reliability = 0.79), discriminant validity (e.g., successfully differentiated between an ASD and student group), and convergent validity with other measures that have been used to assess autistic traits. Participants respond on a 4‐point Likert‐type scale with 0 = false, not at all; 1 = slightly true; 2 = mainly true; and 3 = very true. The 24 items are shown in the Supporting Information: Appendix A.

The CRT‐Long (Primi et al. 2016) is used to measure cognitive reflection. The CRT‐L problems used in the current study, with the analytic (correct) and intuitive responses, are shown in the Supporting Information: Appendix B. After completing the CRT‐L, participants are asked to estimate how many of the test questions (out of 6) they think they answered correctly and then to compare their ability to answer the questions correctly with that of the average person by providing a percentile ranking. Participants are provided with an explanation that percentile rankings can range from 0 (“I'm at the very bottom”) to 50 (“I'm exactly average”) to 99 (“I'm at the very top”).

## 3 Results

As indicated in Table 1, our autistic participants gave significantly fewer intuitively appealing incorrect responses than non‐autistic participants (*t* = −2.05, *p* = 0.04, *d* = 0.21). In addition, the autistic participants provided more correct (analytic) answers (*M* = 3.4, SD = 2.1) than the non‐autistic participants (*M* = 2.7, SD = 2.1). However, this difference was not significant. A correlation matrix on all study variables, including demographic variables, for autistic and non‐autistic participants, is provided in the Supporting Information: Appendix C, Table S1.

As expected, participants overestimated their accuracy on the 6 CRT items. On average, they believed they had correctly solved 4.31 CRT problems (SD = 1.65), whereas their actual mean performance was only 3.01 (SD = 2.09). The Spearman's correlation between estimated and actual CRT performance was moderate, *r* = 0.38, *p* < 0.001, indicating that actual CRT performance accounted for just 14.4% of the variance in estimated performance.

**FIGURE 1:**
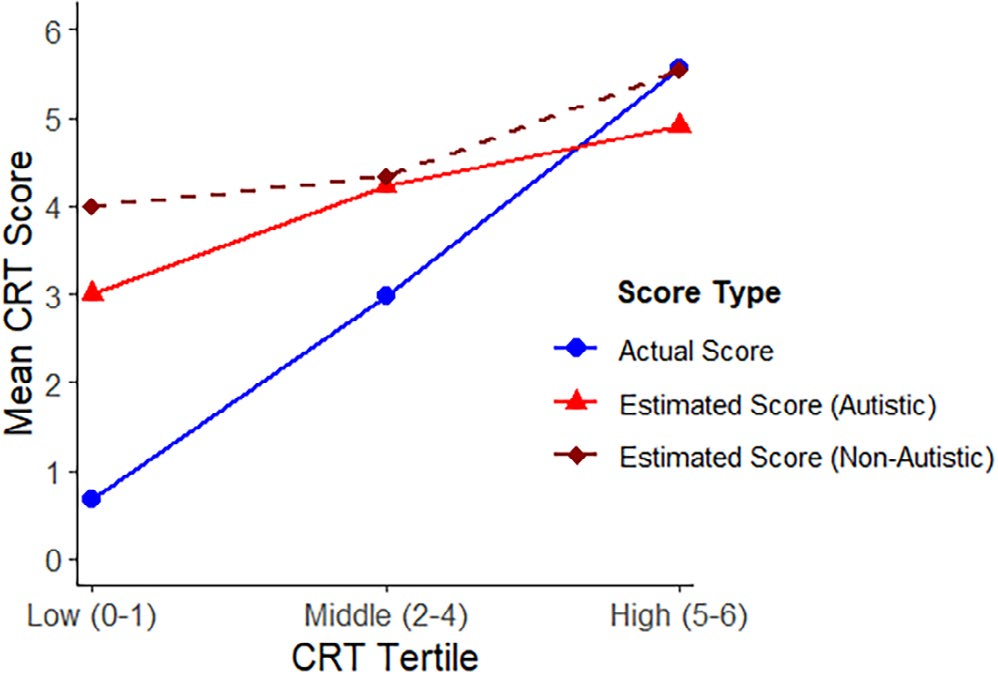
Estimated and actual CRT scores for autistic and non‐autistic participants by tertile.

**FIGURE 2:**
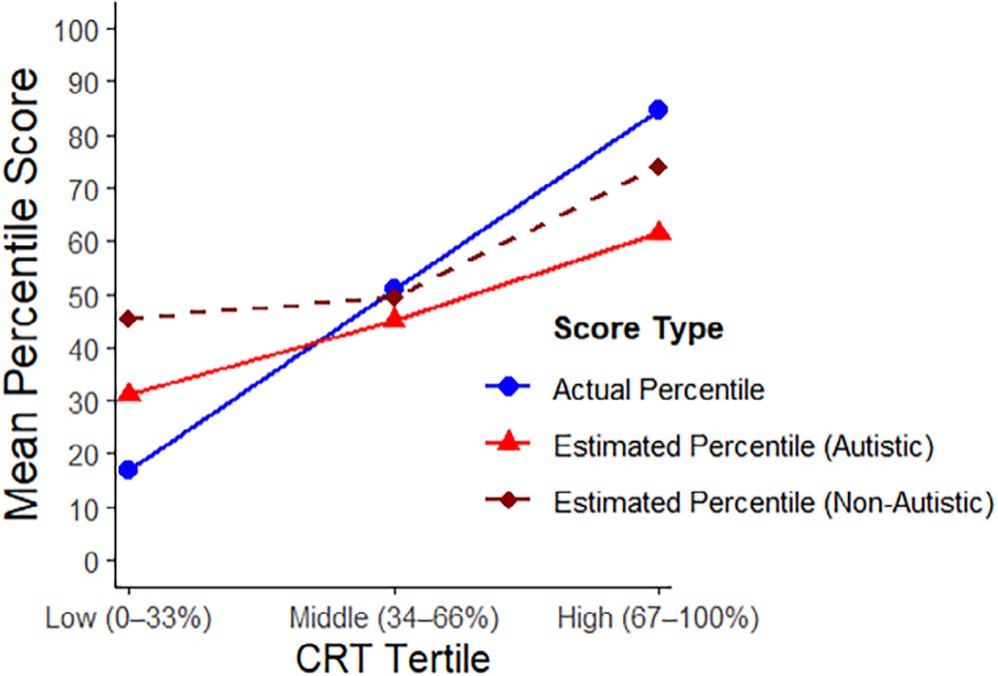
Estimated percentile and actual percentile for autistic and non‐autistic participants by tertile.

To further investigate, we followed the approach of Kruger and Dunning (1999) and divided the sample into performance subgroups based on accuracy. This allowed us to examine whether participants with lower scores on the CRT were more miscalibrated than those with higher scores. Although the six‐item CRT theoretically permits seven distinct performance groups, the sample sizes for some scores were too small for meaningful analysis (e.g., 0: *N* = 11; 3: *N* = 8). To address this, we adopt the post hoc method used by other investigators who have studied the DKE in the context of performance on the CRT (e.g., Pennycook et al. 2017) by combining adjacent scores to form three broader groups (“low”: scores of 0–1, “middle”: scores of 2–4; and “high”: scores of 5–6). This allows more participants per subgroup without altering the overall patterns observed. A chi‐square test indicated that the distribution of autistic and non‐autistic participants across the three groups or tertiles was not significantly different ($x^{2}$ = 3.07, df = 2, *p* = 0.21, *V* = 0.10). Figure 1 shows the average actual test scores and average estimated test scores for autistic and non‐autistic participants in each tertile. Figure 2 shows the actual percentiles and the estimated percentiles for autistic and non‐autistic participants by tertile.

As is evident from Figure 1, these results mirror the pattern typically observed in studies of the DKE. For autistic and non‐autistic participants, overestimation decreased systematically as accuracy increased. In Figure 1, incompetent participants, defined as those who scored in the low tertile, overestimated their performance to a greater degree than those in the middle and high groups. In Figure 2, participants in the low tertile overestimated their percentile score, whereas participants in the high tertile, especially autistic participants, tended to underestimate their ability relative to their peers. Participants in the middle tertile were less miscalibrated.

**FIGURE 3:**
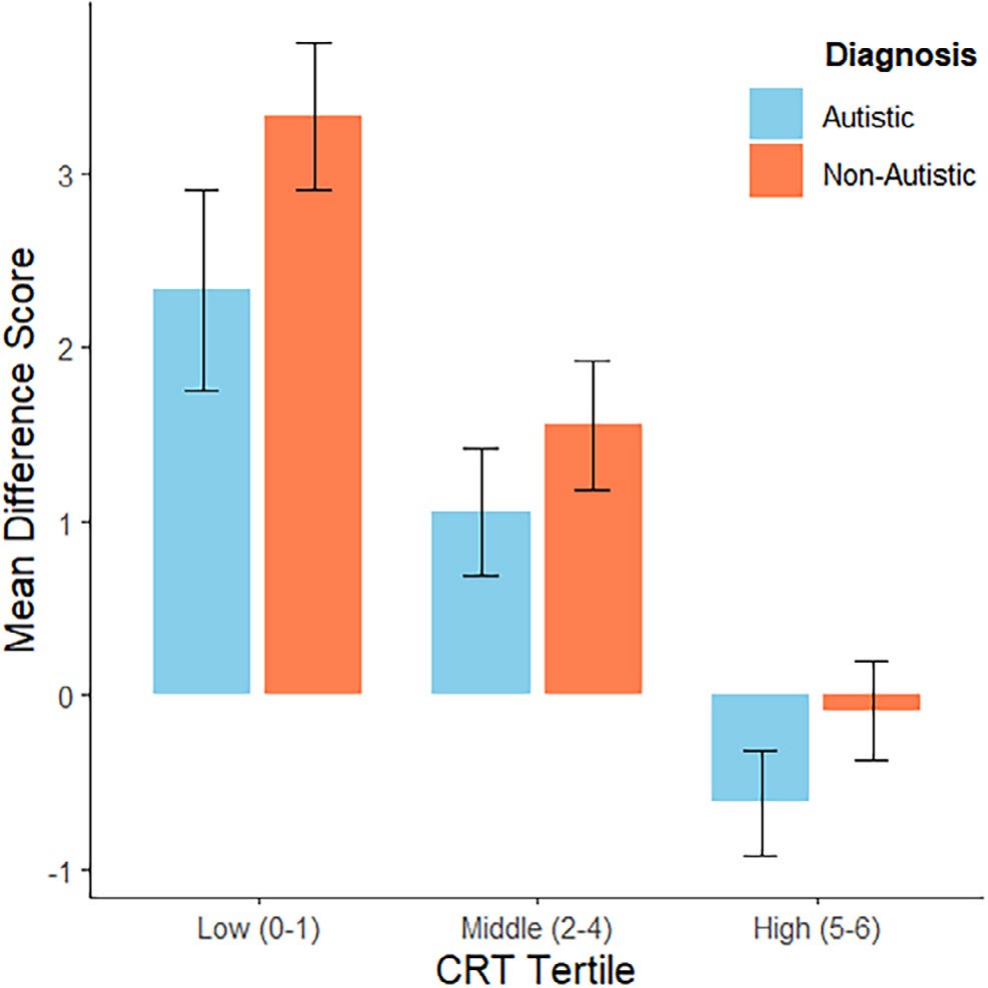
CRT difference scores for autistic and non‐autistic participants by tertile (error bars represent ±1 standard deviation).

**FIGURE 4:**
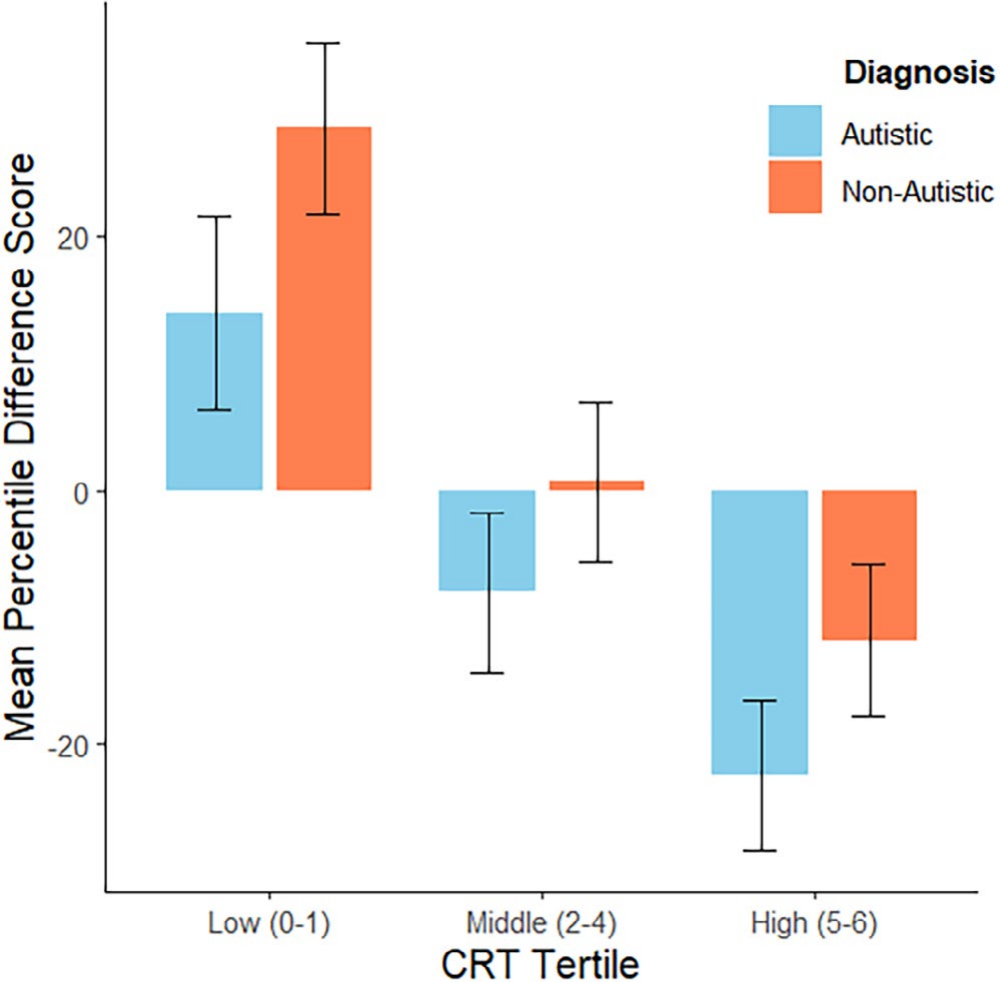
CRT percentile differences for autistic and non‐autistic participants by tertile (error bars represent ±1 standard deviation).

To investigate whether calibration was better for autistic participants than non‐autistic participants, we computed the difference between the estimated and actual CRT score for each individual participant. Positive difference scores represent overestimations, negative are underestimations. The average difference scores for autistic and non‐autistic participants in each tertile are shown in Figure 3. The distribution of difference scores by diagnosis is shown in Supporting Information: Appendix C, Figure S3. We also computed the difference between the estimated and actual CRT percentiles for each individual participant. The average percentile differences for autistic and non‐autistic participants by tertile are shown in Figure 4. Means and standard deviations of the actual, estimated, and difference scores for autistic and non‐autistic participants by tertile are shown in Supporting Information: Appendix C, Table S2. Means and standard deviations of the actual, estimated, and percentile differences for autistic and non‐autistic participants by tertile are shown in Supporting Information: Appendix C, Table S3.

A two‐way ANOVA^5^ of the CRT difference scores for autistic and non‐autistic participants across the three tertiles revealed a significant main effect of diagnosis, *F*(1, 94) = 11.23, *p* = 0.001, *η* ^2^ = 0.11, indicating that autistic participants underestimated their performance overall compared to non‐autistic participants. Additionally, there was a significant main effect of CRT tertile, *F*(2, 94) = 29.99, *p* ≤ 0.001, *η* ^2^ = 0.39, confirming that overestimation decreased as accuracy increased for both groups. However, the interaction between diagnosis and tertile was not statistically significant, *F*(2, 94) = 0.24, *p* = 0.78, *η* ^2^ ≤ 0.001. Tukey's HSD post hoc analyses showed that participants in the low tertile consistently overestimated their scores compared to those in the high tertile, regardless of diagnosis. Tukey's HSD allows multiple comparisons to adjust for familywise error rate, allowing pairwise contrasts between all tertile levels while controlling for Type I error. Non‐autistic participants exhibited greater overestimation than autistic participants, particularly in the low and middle tertiles. For instance, the difference in CRT estimation scores between non‐autistic participants in the low tertile and autistic participants in the high tertile was statistically significant, with a mean difference of 3.95 (SE = 0.52), *t*(94) = 7.54, *p* < 0.0001. Moreover, the difference between autistic participants in the low and high tertiles was also statistically significant (mean difference = 2.95, *p* < 0.001). Finally, non‐autistic participants in the low tertile significantly overestimated their performance compared to non‐autistic participants in the high tertile (mean difference = 3.42, SE = 0.63, *t*(94) = 5.48, *p* < 0.0001). These findings suggest that participants with lower actual scores had poorer self‐estimates of their performance, regardless of diagnostic status.

Similarly, a two‐way ANOVA of the CRT percentile differences for autistic and non‐autistic participants across the three tertiles revealed a significant main effect of diagnosis, *F*(1, 94) = 8.77, *p* = 0.004, *η* ^2^ = 0.09, indicating less overestimation in autistic participants. Additionally, there was a significant main effect of CRT tertile, *F(*2, 94) = 16.89, *p* < 0.001, *η* ^2^ = 0.26, confirming that overestimation decreased as analytic reasoning increased. However, the interaction between diagnosis and tertile was not statistically significant, *F*(2, 94) = 0.10, *p* = 0.90, *η* ^2^ ≤ 0.001. Tukey's HSD post hoc analyses revealed that participants in the low tertile consistently overestimated their percentile scores compared to the high tertile, regardless of diagnosis. Specifically, autistic participants in the low tertile significantly overestimated their percentile scores relative to autistic participants in the high tertile (mean difference = 36.49, SE = 9.08, *t* (94) = 4.02, *p* = 0.0016). Non‐autistic participants in the low tertile also significantly overestimated their scores compared to their high‐tertile counterparts (mean difference = 40.33, SE = 10.30, *t* (94) = 3.92, *p* = 0.0022). The largest effect was observed between non‐autistic participants in the low tertile and autistic participants in the high tertile, with a mean difference of 51.01 (SE = 8.63, *t* (94) = 5.91, *p* < 0.0001). Additionally, non‐autistic participants exhibited slightly greater overestimation than autistic participants across all tertiles, although differences between groups within tertiles were not statistically significant (all *p* values > 0.63). These results further support that overestimation bias was most pronounced among low scorers and particularly elevated in non‐autistic participants.

## 4 Discussion

Despite numerous studies investigating the relationship between autism and metacognition, the evidence remains mixed (Carpenter and Williams 2023). This study measured metacognition as the correspondence between estimated and actual performance in a cognitive reflection task. Results indicated asymmetric calibration of actual versus estimated CRT performance in autistic employees: In the low‐performance group, autistic participants overestimated their abilities less than non‐autistic participants, but in the high‐performance group, autistic participants underestimated their abilities more than non‐autistic participants. Theoretical and practical implications of these results are highlighted.

### 4.1 Theoretical Implications

The purpose of this study was not to investigate differences between autistic and non‐autistic individuals in performance on the CRT. That research is ancillary to this study. However, consistent with other studies (e.g., Brosnan and Ashwin 2023), our autistic participants gave fewer intuitive and a higher proportion of analytic responses than our non‐autistic participants. But we did not match our comparison groups on cognitive ability, which some researchers have suggested is related to performance on the CRT (e.g., Morsanyi and Hamilton 2023).

The CRT does have moderate overlap with measures of cognitive ability, including reported correlations of 0.44 (Frederick 2005), 0.43 (Obrecht et al. 2009), and 0.40 (Toplak et al. 2011). However, the task also involves thinking dispositions, particularly those related to reflectivity. The one study that explicitly examined this issue (Toplak et al. 2011) concluded that the CRT predicted rational thinking performance independent of intelligence. In other words, the CRT accounted for more unique variance than intelligence measures. Other investigators have also reported that the CRT predicts incremental variance beyond that explained by intelligence in tasks including decision‐making (Baron et al. 2015; Liberali et al. 2012; Primi et al. 2016), financial literacy (Gignac and Stevens 2024), and motivated reasoning (Trippas et al. 2015), supporting its validity as an independent psychological construct. In this research, dispositions are construed as high‐level attitudes and beliefs that regulate how individuals prefer to use their cognitive capacities (Baron et al. 2023; Stanovich 2009).

Evidence on whether autistic individuals exhibit altered metacognition is mixed. Some studies have found differences between autistic and non‐autistic participants (Grainger et al. 2016a; Nicholson et al. 2019; Nicholson et al. 2020; Wilkinson et al. 2010; van der Plas et al. 2021) while other studies report no differences (Embon et al. 2023; Grainger et al. 2016b; Maras et al. 2017, 2020; Sawyer et al. 2014; Wojcik et al. 2011, 2014) and other studies found mixed results (Carpenter et al. 2019; Williams et al. 2018). Fleming and Lau (2014) have proposed that studies reporting no differences between autistic and non‐autistic participants did not quantify metacognition in a way that differentiates between metacognitive sensitivity (defined as the ability to distinguish between correct and incorrect answers) and metacognitive bias (the overall confidence in one's answers). However, studies using bias‐free measures of metacognition have also produced mixed results, with some showing differences between autistic and non‐autistic participants (Nicholson et al. 2020; van der Plas et al. 2021) and some showing no differences (Embon et al. 2023). Our findings complicate the picture but do not support a global advantage in metacognition in autism. Instead, they suggest a measure‐dependent pattern: under the task conditions used here, autistic participants showed relatively greater discrimination between correct and incorrect responses (metacognitive sensitivity) while showing similar or different patterns on overall confidence (metacognitive bias/calibration). Because sensitivity and bias index distinct cognitive processes (and because JOL, FOK and JOC tap different temporal points and cues in monitoring), these results should be interpreted cautiously and in reference to the specific metacognitive measure employed (Koriat 1997).

Accordingly, our primary result, that autistic people may demonstrate higher levels of metacognitive accuracy than non‐autistic people when the measure of metacognition includes both metacognitive sensitivity and metacognitive bias, must be understood in the light of well‐established theoretical distinctions in the metacognition literature. Nelson and Narens' (1990) object‐level/meta‐level framework highlights that monitoring and control are separable processes, and Koriat's (1997) cue‐utilization account stresses that different metacognitive judgments draw on different classes of cues (intrinsic, extrinsic, mnemonic), producing distinct patterns across JOL, FOK and confidence paradigms. Recent autism research likewise emphasizes heterogeneous, measure‐dependent findings (e.g., meta‐analytic heterogeneity and adult vs. child differences), reminding us to avoid sweeping claims about “better” or “worse” metacognition in autism. We therefore frame our contribution as evidence for a conditional advantage on a specific form of monitoring that invites direct comparison across JOL, FOK, and JOC paradigms and replication in larger adult samples.

Studies on the DKE, by definition, do not separate the contributions of metacognitive sensitivity and bias. As a result, “overconfidence” effects can reflect either a general tendency toward high confidence (bias) or a failure to discriminate correct from incorrect performance (sensitivity). This distinction is critical because the original “dual burden” account of the DKE (Kruger and Dunning 1999) aligns more closely with a sensitivity deficit: poor performers not only lack task skill but also show impaired monitoring sensitivity, making it difficult to recognize their errors. In contrast, higher performers possess both stronger task skills and greater metacognitive sensitivity, enabling better‐calibrated self‐assessments. Our results support this interpretation, as we observed the predicted pattern of poor performers showing reduced calibration and high performers demonstrating more accurate confidence judgments, consistent with sensitivity‐driven differences rather than bias alone.

Competing accounts of the DKE focus on other mechanisms that might explain this effect. Some investigators have argued that the overconfidence displayed by the unskilled is a statistical or methodological artifact, for example, regression to the mean (Gignac and Zajenkowski 2020; Krueger and Mueller 2002).^6^ In this study, however, our autistic participants were better calibrated to the objective criterion than our non‐autistic participants. Accordingly, the metacognitive differences between autistic and non‐autistic participants in this study cannot be attributed to a statistical artifact because otherwise the magnitude of misestimates would be the same in both groups. The results reported here are therefore closely aligned with Kruger and Dunning's (1999) original account of a metacognitive deficit among the unskilled.

### 4.2 Practical Implications

The present findings illustrate the everyday consequences of metacognitive differences in analytic thinking. Specifically, less analytic thinkers are less effective at metacognitive monitoring when given a reasoning task and are less accurate at self‐reporting their relative level of analytic thinking. Those most likely to lack competency are also the least likely to recognize their incompetence. In the workplace context, mistakes or poor performance often occur when employees overestimate their skills (e.g., Cheng et al. 2021). In addition, employees who believe they are already highly competent may avoid seeking feedback, training, or mentorship, thus slowing their professional growth (e.g., Yehuda et al. 2024). Individuals in positions of authority may make decisions despite being uninformed about the subject matter, unaware of relevant circumstances or critical events, or project a false sense of competence (Cohee and Barnhart 2024; Huang 2013). Many organizational change initiatives fall short of expectations or fail because executives overestimate their competence and their organization's capabilities, a perspective not always shared by the workers (Nold and Michel 2023).

Given the potential adverse consequences of the DKE in the workplace, mitigation strategies for counteracting biased self‐assessment warrant attention. Educating people about the DKE may not necessarily help them reflect on their competencies. Most humans recognize others may fall prey to such biases but believe they are somehow immune to them (Hartman in press). However, clear performance benchmarks help human learners calibrate their self‐assessments against objective benchmarks when provided with regular and constructive feedback on performance (e.g., Giamos et al. 2023). For example, students in the classroom benefit from regular performance feedback or tests that can provide objective insights into their skill levels (Lipnevich and Smith 2009). Similarly, employees in the workplace benefit from regular and constructive feedback on their performance (Tseng and Levy 2019). In addition, teaching individuals to reflect on their learning and evaluate errors can improve self‐awareness (Lew and Schmidt 2011). Finally, creating a culture that normalizes seeking and receiving feedback can help employees recognize their limitations (Hartman in press). Fostering a culture of accountability and open communication can create more honest and resilient environments that facilitate more successful employment experiences for all personnel, not just autistic employees (Hartman and Hartman 2024b).

### 4.3 Field Research on the DKE in the Workplace

Finally, there is a need for field research to explore the implications of the DKE in the workplace. Experimental studies can be conducted on many different facets of the DKE in the workplace and at multiple levels of analysis, including individual, team, and organizational. For example, 360‐degree surveys provide estimates of how leaders assess their own effectiveness in demonstrating competencies, which can be calibrated against how others evaluate the leader's effectiveness in those domains. Do leaders demonstrate more accurate self‐awareness or seek more input from others when they receive feedback from 360‐degree surveys? What is the relationship between personality traits (e.g., openness to experience) and the DKE? Is there better calibration of self‐assessments when employees are provided with clear performance benchmarks or when they receive regular, constructive feedback? In making hiring decisions where candidates may overstate their skills during interviews, do behavioral simulations help recruiters differentiate between perceived and actual competence? Does an organizational culture that fosters a “growth mindset,” where learning and development are prioritized over short‐term performance, counteract the negative effects of the DKE?

### 4.4 Autistic Employees and the DKE

Employers are looking for viable pathways to address diversity‐related issues and leverage the inherent potential of a diverse workforce. Interest in organizational neurodiversity, especially, is increasing (Doyle 2020). This study contributes to a growing body of empirical evidence highlighting potential advantages of autistic employees in the workplace. While employees who are poor performers are not likely to recognize that they are doing badly, autistic employees are less susceptible to such errors in self‐assessment. These findings provide valuable insights into both the cognitive strengths of autistic individuals and the mechanisms underlying metacognitive biases.

The results of this study also have implications for human resource practices in hiring, onboarding, and managing autistic employees. For example, not only are simulations perceived more favorably than job interviews by autistic job candidates (de Vries 2024), but these findings also help to explain why hiring decisions based on job interviews (which are influenced by social style, impression management, and the DKE) are biased against autistic job candidates (Maras et al. 2021). This is particularly the case with unstructured interviews as opposed to structured interviews, where candidates are asked a consistent set of questions with clear criteria to assess the quality of responses. In addition, there is a need for research to elucidate how these findings shed light on performance appraisal, feedback, and coaching processes for autistic employees. For example, these results suggest that autistic employees are likely to be more open to acknowledging skills in need of improvement and less likely to acknowledge their areas of strength. How does that change the frame of reference for a manager conducting a performance appraisal with an autistic employee? These are only a few of the many questions that need to be investigated in this new and fertile arena for personnel research.

### 4.5 Limitations and Future Research Directions

The relationship between autism and metacognition may vary across specific subpopulations of autistic people. For example, our sample of autistic participants was employees working full‐time or part‐time. Evidence indicates that autistic individuals with higher levels of social competence are more likely to achieve employment and live independently (Clarke and Lord 2024). However, 85%–90% of autistic adults are unemployed or severely underemployed (Howlin 2013), and these rates only improve to 75% for those with a university degree (Hurley‐Hanson and Giannantonio 2016). The subpopulation of unemployed autistic adults was not represented in our sample (only one of our 53 autistic participants was unemployed at the time of the study). It is possible that studies with a different subpopulation of autistic people, those who are not able to obtain and maintain employment, may not find higher levels of metacognitive sensitivity.

Another possibility is that the manifestation of the DKE may vary across domains or tasks. The DKE has been observed in cognitive, social, and emotional domains (Ernst et al. 2023). For example, the original Kruger and Dunning (1999) study used tasks involving humor, logic, and grammar to compare actual performance to estimated performance. Some domains of competence, like the one examined here (analytic thinking), are those in which autistic individuals may have an advantage (Brosnan and Ashwin 2023). In other domains, however, competency depends on different factors, such as understanding social communication, where autistic individuals are less skilled (American Psychiatric Association 2013). Future studies should consider differences in the DKE between autistic and non‐autistic individuals using tasks that focus on social and emotional domains to ensure the generalizability of reduced susceptibility to the DKE in autistic adults.

A third limitation of this study is that our two groups, autistic and non‐autistic, were different not just in terms of diagnosis, but also in age. Our autistic participants' average age was 32.2 versus 38.7 for our non‐autistic participants (*t* = −2.63, *p* = 0.01, *d* = 0.27). Although research shows that CRT scores are stable over time (e.g., Stagnaro et al. 2013), future research with better matching of autistic and non‐autistic participants on demographic variables is warranted.

### 4.6 Conclusion

In summary, these findings suggest that autistic employees are less likely to make errors in self‐assessments of their cognitive performance than non‐autistic employees. As a result of their reduced susceptibility to the DKE, autistic employees may contribute to improvements in decision‐making, enhanced collaboration, increased innovation, improved leadership effectiveness, and a stronger organizational culture, leading to better strategy execution, customer satisfaction, and financial results.

## Ethics Statement

This study received approval from the York University Ethics Review Committee (HPRS Certificate #: e2024–078).

## Conflicts of Interest

The authors declare no conflicts of interest.

## Supporting information


**Data S1:** Supporting Information.

## Data Availability

The data that support the findings of this study are available on request from the corresponding author. The data are not publicly available due to privacy or ethical restrictions.

## Endnotes

^1^
We use “identity first” language to respect the views of many autistic people and their families (e.g., Kenny et al. 2016). Also, acknowledging the wider natural variation of neurodiversity, we use the term “non‐autistic” rather than “neurotypical” or “typically developing” to describe people who do not have a diagnosis of autism.

^2^
An anonymous reviewer suggested it is “commendable that the paper emphasizes the strengths of autistic individuals—a valuable and progressive step forward—but this strengths‐based focus can risk obscuring core challenges and may inadvertently limit access to crucial support services that many autistic people still need. This important nuance should be more clearly reflected in the manuscript to ensure a balanced perspective that acknowledges both strengths and the ongoing need for support in areas such as social cognition and workplace inclusion.” The reviewer's point is well‐taken. While research highlights the cognitive strengths of autistic individuals, such as reduced susceptibility to certain biases and social pressures, it is equally important to recognize that these traits can also present challenges, especially in the domain of social cognition. Framing characteristics as “advantages,” like being less influenced by social norms, reputation concerns, or others' intentions, may also reflect genuine difficulties in interpreting or navigating complex social situations. These differences are not value‐neutral; they often represent core challenges in social communication that can complicate workplace integration. For example, reduced responsiveness to social influence or moral intention might lead to misunderstandings, perceptions of insensitivity, or friction in team dynamics. Skills such as indirect communication, reading subtle cues, and adapting to unspoken expectations, often critical in workplace cultures, may not come intuitively to autistic employees. Without explicit support and guidance, these gaps can contribute to social exclusion, isolation, or even discrimination, regardless of strong technical or job‐specific performance. Thus, while it is vital to acknowledge and celebrate strengths, it is just as crucial not to minimize the real social vulnerabilities autistic individuals may encounter in employment settings.

^3^
Social desirability biases may operate differently across the autistic spectrum. For example, it is commonly believed that sex differences in diagnostic prevalence may reflect the fact that autistic women are more likely to engage in camouflaging behaviors to conform socially, often driven by a strong desire for acceptance and belonging. Efforts to appear competent or socially appropriate may explain the later age of diagnosis in autistic females compared to males. However, evidence on whether masking (or camouflaging) is more prevalent among autistic females and gender‐diverse individuals is mixed. Some researchers have found that relatively equal numbers of autistic males and females, and all individuals of other genders, report camouflaging, with no patterns of differences in camouflaging behaviors between males and females (Hull et al. 2017). However, in a more recent review of the literature, these researchers have suggested that autistic females appear to demonstrate more camouflaging than autistic males (Cook et al. 2021). Similarly, in a study using the CAT‐Q (a quantitative self‐report questionnaire on camouflaging), researchers found autistic females scored significantly higher than autistic males (Hull et al. 2019); however, these sex differences have not been replicated across all studies using the CAT‐Q (e.g., Cage and Troxell‐Whitman 2019). Future research to explore how social desirability biases operate differently across the spectrum would be interesting.

^4^
The one autistic participant who reported being unemployed at the time of the study had recently been laid off after working for 7 years with the same employer. Of the three non‐autistic participants who reported being unemployed at the time of the study, one was on disability leave, and two were unpaid volunteers working half‐time for a disability support agency.

^5^
ANOVA is the standard statistical method for comparing the means of multiple groups and their interactions. An ANOVA is the most appropriate test to assess the effect of Diagnosis (autistic vs. non‐autistic) and CRT performance tertile (low, medium, high) on the continuous dependent variable (the difference score) and their interaction. Multiple *T‐tests* wouldn't be appropriate because it would dramatically inflate the risk of a Type I error, due to multiple comparisons. Regression would also be less appropriate because DKE describes a non‐linear relationship, where the degree of overestimation changes based on skill level. Furthermore, ANOVA is a common and appropriate statistical tool used in studies of the DKE (e.g., Morsanyi and Hamilton 2023).

^6^
“Krueger,” not to be confused with “Kruger.”
