# Supplementary material for: Reduced Susceptibility to the Dunning–Kruger Effect in Autistic Employees
Source: Autism Res. 2025 Nov 12;19(1):e70139. doi: 10.1002/aur.70139 (PMC12853236; doi:10.1002/aur.70139)
Supplement: Supplementary file 1 — Data S1: Supporting Information. [file AUR-19-0-s001.docx]

Supplementary Materials- Appendix A

The Subthreshold Autistic Trait Questionnaire (SATQ; Kanne et al., 2012)

This is a 24-item self-report questionnaire with four possible responses. Participants are told "For each item, please use the scale below to rate the extent to which it describes you on most days. There are no right or wrong answers. Please answer all of the items the best you can." They respond on a 4-point Likert-type scale with 0=false, not at all; 1 = slightly true; 2 = mainly true; and 3 = very true. The 24 items are shown below. To avoid a response bias, 10 questions are worded such that higher scores represent higher autistic traits, and 14 are worded such that higher scores represent lower autistic traits (these items, designated with an ®, are reverse coded):

1. I like being around other people. ®

2. I enjoy social situations where I can meet new people and chat (i.e., parties, dances, sports, games). ®

3. I seek out and approach others for social interactions. ®

4. I like to share my enjoyment with others. ®

5. Others consider me warm, caring, and/or friendly. ®

6.I respond appropriately to other people’s emotions (for example, comforting someone who is upset). ®

7.I can have a back-and-forth conversation (listen well and change topics appropriately). ®

8. I use many gestures when speaking with others such as shrugging, ‘‘talking with my hands,’’ nodding my head, etc.

9. Others think that I am strange or bizarre.

10. I have some behaviors that others consider strange or odd.

11. I sometimes say things that others tell me are rude or inappropriate.

12. I use odd phrases or tend to repeat certain words or phrases over and over again.

13. I am very interested in things related to numbers (i.e. dates, phone numbers, etc.).

14. I am good at knowing what others are feeling by watching their facial expressions or listening to the tone of their voice. ®

15.I can sense that someone is not interested in what I’m saying by reading their facial expressions. ®

16. I make eye contact when talking with others. ®

17.I am good at using words to express my thoughts and ideas. ®

18.I have difficulty getting my ideas across to others in a conversation.

19.I have a good imagination. ®

20. I am comfortable with spontaneity, such as going to new places and trying new things. ®

21. I tend to stick to routines in my day-to-day life, preferring to do things the same way.

22 .I am considered ‘‘laid back’’ and am able to ‘‘go with the flow’’. ®

23. I sometimes take things too literally, such as missing the point of a joke or having trouble understanding sarcasm.

24. I tend to focus on individual parts and details more than the big picture.

Supplementary Materials-Appendix B

The CRT-Long (Primi et al., 2016)

The CRT-Long (Primi et al., 2016) is used to measure cognitive reflection. The CRT-L problems used in the current study, with the typical analytic and intuitive responses, are:

(1) A bat and a ball cost $1.10. The bat costs $1.00 more than the ball. How much does the ball cost? [analytic reponse = 5 cents; intuitive response = 10 cents].

(2) If it takes 5 min for five machines to make five widgets, how long would it take for

100 machines to make 100 widgets? [analytic response = 5 min; intuitive

response = 100 min]

(3). In a lake, there is a patch of lily pads. Every day, the patch doubles in size. If it takes 48 days for the patch to cover the entire lake, how long would it take for the patch to cover half of the lake? [analytic response = 47 days; intuitive response = 24 days]

(4). If three elves can wrap three toys in 1 h, how many elves are needed to wrap six toys in 2 h? [analytic response = 3 elves; intuitive response = 6 elves]

(5). Jerry received both the 15th highest and the 15th lowest mark in the class. How

many students are there in the class? [analytic response = 29 students; intuitive

response = 30 students]

(6). In an athletics team, tall members are three times more likely to win a medal than

short members. This year the team has won 60 medals so far. How many of

these have been won by short athletes? [analytic response = 15 medals; intuitive

response = 20 medals]

Supplementary Materials – Appendix C

**Figure 1. Distribution of SATQ Scores by Diagnosis**


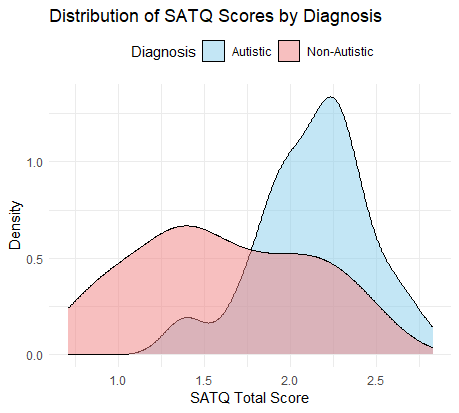


**Figure 2. Distribution of CRT Scores by Diagnosis**


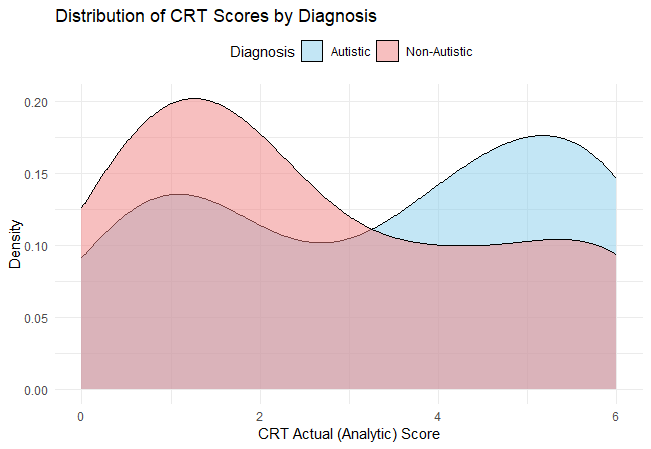


**Figure 3. Distribution of CRT Difference Scores by Diagnosis**


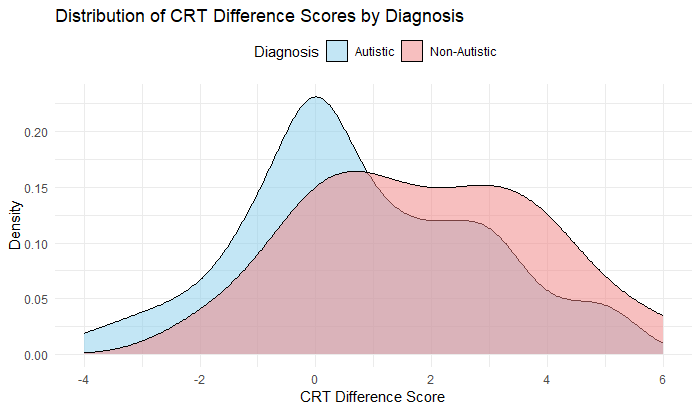


**Table 1. Means, standard deviations, and correltions with confidence intervals**

***Means, standard deviations, and correlations with confidence intervals***

| **Variable** | ***M*** | ***SD*** | **1** | **2** | **3** | **4** | **5** | **6** | **7** |
| --- | --- | --- | --- | --- | --- | --- | --- | --- | --- |
|  |  |  |  |  |  |  |  |  |  |
| **1. Age** | **35.64** | **12.81** |  |  |  |  |  |  |  |
|  |  |  |  |  |  |  |  |  |  |
| **2. Sex** | **1.72** | **0.45** | **.05** |  |  |  |  |  |  |
|  |  |  | **[-.15, .25]** |  |  |  |  |  |  |
|  |  |  |  |  |  |  |  |  |  |
| **3. Education** | **3.29** | **1.20** | **.17** | **.11** |  |  |  |  |  |
|  |  |  | **[-.03, .35]** | **[-.08, .30]** |  |  |  |  |  |
|  |  |  |  |  |  |  |  |  |  |
| **4. Employment** | **1.78** | **0.95** | **-.00** | **-.15** | **-.21*** |  |  |  |  |
|  |  |  | **[-.20, .19]** | **[-.33, .05]** | **[-.39, -.01]** |  |  |  |  |
|  |  |  |  |  |  |  |  |  |  |
| **5. SATQ** | **1.89** | **0.49** | **-.22*** | **.01** | **-.02** | **.08** |  |  |  |
|  |  |  | **[-.40, -.02]** | **[-.19, .20]** | **[-.22, .18]** | **[-.12, .27]** |  |  |  |
|  |  |  |  |  |  |  |  |  |  |
| **6. CRT: Analytical** | **3.04** | **2.09** | **-.12** | **.00** | **-.14** | **.06** | **.21*** |  |  |
|  |  |  | **[-.31, .08]** | **[-.20, .20]** | **[-.33, .06]** | **[-.14, .25]** | **[.01, .39]** |  |  |
|  |  |  |  |  |  |  |  |  |  |
| **7. CRT: Intuitive** | **2.09** | **1.85** | **.17** | **.04** | **.11** | **-.01** | **-.25*** | **-.86**** |  |
|  |  |  | **[-.03, .35]** | **[-.16, .24]** | **[-.09, .30]** | **[-.20, .19]** | **[-.43, -.06]** | **[-.91, -.80]** |  |
|  |  |  |  |  |  |  |  |  |  |
| **8. CRT: Atypical** | **0.87** | **1.06** | **-.06** | **-.08** | **.09** | **-.10** | **.03** | **-.47**** | **-.05** |
|  |  |  | **[-.25, .14]** | **[-.27, .12]** | **[-.11, .28]** | **[-.29, .10]** | **[-.16, .23]** | **[-.61, -.30]** | **[-.24, .15]** |
|  |  |  |  |  |  |  |  |  |  |

***Note.* *M* and *SD* are used to represent mean and standard deviation, respectively. Values in square brackets indicate the 95% confidence interval for each correlation. The confidence interval is a plausible range of population correlations that could have caused the sample correlation (Cumming, 2014). * indicates *p* < .05. ** indicates *p* < .01.**

**Table 2. Actual, Estimated, and Difference Scores for Autistic and Non-Autistic Participants by Tertile**

Low Middle High

Autistic Non-Autistic Autistic Non-Autistic Autistic Non-Autistic

*N*  15 18 17 17 21 12

Estimated 3.00 (2.04) 4.00 (1.78) 4.24 (1.15) 4.33 (1.53) 4.90 (1.41) 5.55 (0.69)

Actual 0.67 (0.49) 0.67 (0.49) 3.18 (0.88) 2.78 (0.88) 5.52 (0.51) 5.64 (0.50)

Difference 2.33 (2.23) 3.33 (1.78) 1.06 (1.52) 1.56 (1.58) -0.62 (1.40) -0.09 (0.94)

Means (Standard Deviations)

**Table 3. Actual, Estimated, and Percentiles Differences for Autistic and Non-Autistic Participants by Tertile**

Low Middle High

Autistic Non-Autistic Autistic Non-Autistic Autistic Non-Autistic

*N*  15 18 17 17 21 12

Estimated 31.03 (27.58) 45.56 (28.50) 45.21 (23.76) 49.61 (26.98) 61.43 (27.41) 73.91 (16.53)

Actual 17.00 (8.05) 17.00 (8.00) 53.21 (9.48) 48.92 (9.46) 83.88 (8.19) 85.68 (8.07)

Difference 14.03 (29.49) 28.56 (28.64) -8.00 (26.03) 0.69 (26.82) -22.45 (27.02) -11.77 (20.0)

Means (Standard Deviations)
